# Involvement of SNPs in miR-3117 and miR-3689d2 in childhood acute lymphoblastic leukemia risk

**DOI:** 10.18632/oncotarget.25144

**Published:** 2018-05-01

**Authors:** Angela Gutierrez-Camino, Idoia Martin-Guerrero, Vita Dolzan, Janez Jazbec, Ana Carbone-Bañeres, Nagore Garcia de Andoin, Ana Sastre, Itziar Astigarraga, Aurora Navajas, Africa Garcia-Orad

**Affiliations:** ^1^ Department of Genetics, Physical Anthropology and Animal Physiology, University of the Basque Country, UPV/EHU, Leioa, Spain; ^2^ Institute of Biochemistry, Faculty of Medicine, Ljubljana, Slovenia; ^3^ Department of Oncology and Haematology, University Children’s Hospital, University Medical Centre Ljubljana, Ljubljana, Slovenia; ^4^ Department of Paediatrics, University Hospital Miguel Servet, Zaragoza, Spain; ^5^ Department of Paediatrics, University Hospital Donostia, San Sebastian, Spain; ^6^ BioDonostia Health Research Institute, San Sebastian, Spain; ^7^ Department of Oncohematology, University Hospital La Paz, Madrid, Spain; ^8^ Department of Paediatrics, University Hospital Cruces, Barakaldo, Spain; ^9^ BioCruces Health Research Institute, Barakaldo, Spain

**Keywords:** SNP, miRNAs, acute lymphoblastic leukemia, susceptibility, MAPK signalling pathway

## Abstract

Acute lymphoblastic leukemia (ALL) is the most common cancer in children. Numerous studies have shown that microRNAs (miRNAs) could play a role in this disease. Nowadays, more than 2500 miRNAs have been described, that regulate more than 50% of genes, including those involved in B-cell maturation, differentiation and proliferation. Genetic variants in miRNAs can alter their own levels or function, affecting their target gene expression, and then, may affect ALL risk. Therefore, the aim of this study was to determine the role of miRNA genetic variants in B-ALL susceptibility. We analyzed all variants in pre-miRNAs (MAF > 1%) in two independent cohorts from Spain and Slovenia and inferred their functional effect by *in silico* analysis. SNPs rs12402181 in miR-3117 and rs62571442 in miR-3689d2 were associated with ALL risk in both cohorts, possibly through their effect on MAPK signalling pathway. These SNPs could be novel markers for ALL susceptibility.

## INTRODUCTION

Acute lymphoblastic leukemia (ALL) is the most common childhood malignancy and a leading cause of death due to disease in children [1, 2]. The genetic basis of ALL susceptibility has been supported by its association with certain congenital disorders [3] and, more recently, by several genome-wide association studies (GWAS). These GWAS identified common variants in *ARID5B*, *IKZF1*, *CEBPE* and *CDKN2A* influencing ALL risk in children of European descent [3–8], widely validated [9–14]. While most association studies are mainly focused on the coding regions, which correspond only to 1.5% of the entire genome, many SNPs found in these GWAS are located in non coding regions.

MiRNAs are non-coding RNAs that regulate gene expression at the post-transcriptional level by binding to the 3′ untranslated region (UTR) of a target mRNA, leading to its translation inhibition or degradation [15]. Through this mechanism, miRNAs regulate more than 50% of human genes, having an enormous impact on the function of any cell [16], including B-lymphocytes.

It has been widely shown that miRNAs regulate B-cell maturation and function, controlling B-cell receptor (BCR) signalling, B-cell migration/adhesion, cell–cell interactions in immune niches, and the production and class-switching of immunoglobulins [17, 18]. They also contribute to the regulation of important signalling pathways such as tyrosine kinase and Ras signalling [18], whose deregulation has been demonstrated in ALL [19]. In fact, recent studies have found more than 200 miRNAs deregulated in pediatric patients diagnosed with B-cell precursor acute lymphoblastic leukemia (B-ALL) [20–23]. All these data show the role of miRNAs in pediatric B-ALL.

Genetic variations in miRNAs can alter their function affecting their target genes expression. These variants can modify miRNA expression levels if they are located in the pre-miRNA, or the mRNA-miRNA binding if they are located in the seed region. Nowadays, several works have already described polymorphisms in miRNAs associated with the susceptibility to different types of cancer [24, 25]. Despite all these evidences, only four studies analyzing the involvement of SNPs in miRNAs in the risk of ALL have been performed, and interestingly, all of them found significant findings [26–29]. Hasani and colleagues found rs2910164 in miR-146a associated with ALL susceptibility in a Iranian population of 75 children diagnosed with ALL [26]. Tong and colleagues found association between rs11614913 in miR-196a-2 and rs4938723 in miR-34b and ALL risk in a Chinese population of 574 pediatric ALL patients [27, 29]. Recently, our group found association between rs12803915 in miR-612, rs3746444 in miR-499 and rs10061133 in miR-449b and B-ALL risk in a Spanish cohort of 213 children [28]. Of note is the fact that, although a relatively low number of SNPs in miRNAs were analyzed in relation to B-ALL susceptibility, significant results were found.

Considering all these data and that nowadays the number of annotated miRNAs has increased substantially up to approximately 2500 miRNAs [30], the aim of this study was to determine the role of the currently described SNPs in miRNAs in the risk of B-ALL. For this aim, we analyzed all variants in pre-miRNAs genes with a minor allele frequency higher than 1% in two independent cohorts of Spanish and Slovenian origin. The putative functional implication of significant variants was inferred by *in silico* analysis.

## RESULTS

### Genotyping results

Genotyping analyses were performed in 310 patients with B-ALL (231 from Spain and 79 from Slovenia) and 434 unrelated healthy controls (338 from Spain and 96 from Slovenia). Successful genotyping was achieved for 718 of 744 DNA samples (96.5%), 217 children with B-ALL and 330 controls from the Spanish cohort and 75 children with B-ALL and 96 controls from the Slovenian cohort (Table 1). From the total of 213 SNPs, 135 SNPs (63.4%) were included in the association analysis after eliminating SNPs with genotyping failures (< 80%), monomorphic in our population or with deviations from HWE in controls (Supplementary Table 1). Genotyping was validated using TaqMan OpenArray technology in 175 samples and 15 SNPs and the concordance between both platforms was 99.8% (Supplementary Table 2).

**Table 1:** Study population

	Spanish cohort		Slovenian cohort	
	Patients	Controls	Patients	Controls
**No. of individuals**	231	338	79	96
**Mean age ± SE, y**	4.04 ± 3.61	57.8 ± 28.1	4.65 ± 5.41	44.5 ± 9.4
Sex^*^				
**Males, *n* (%)**	128 (55.7)	157 (46.4)	41 (51.9)	58 (60.4)
**Females, *n* (%)**	102 (44.3)	181 (53.6)	38 (48.1)	38 (39.6)
**Genetic alterations**^#^				
**Hyperdiploid**	56 (24.2)	-	9 (11.4)	-
***ETV6-RUNX1***	37 (16.0)	-	12 (15.2)	-
***MLL***	13 (5.6)	-	4 (5.1)	-
***BCR-ABL***	6 (2.6)	-	1 (1.3)	-
***E2A-PBX1***	6 (2.6)	-	-	-
**Hypodiploid**	2 (0.9)	-	1 (1.3)	-
**Other**	1 (0.4)	-	6 (7.6)	-
**No alteration**	95 (41.1)	-	48 (60.8)	-
**Not available**	21 (9.1)	-	0	-

SE: standard error, y: years ^*^There is no datum for one patient of the Spanish cohort. ^#^Six patients have more than one alteration in the Spanish cohort and two of the patients have more than one alteration in the Slovenian cohort.

### Genotype association study of B-ALL

We analyzed all the SNPs in both populations separately. From the total of 135 SNPs, we found two SNPs in two miRNAs, rs12402181 in miR-3117-3p and rs62571442 in miR-3689d2, significantly associated with B-ALL risk in both Spanish and Slovenian population (Supplementary Table 3). Both SNPs remained significant after multivariate logistic regression to account for the possible confounding effect of sex.

The AA genotype of rs12402181 in miR-3117-3p displayed a 1.44-fold increased risk of B-ALL (95% CI: 1.01–2.08; *p =* 0.047) under the log-additive genetic model (GG vs AG vs AA) in the Spanish population. The same effect was observed in the Slovenian cohort (OR: 2.01; 95% CI: 1.02–3.95; *p =* 0.041). When both populations were analyzed together, they showed the same trend increasing the *p* value (OR: 1.53; 95% CI: 1.12–2.09; *P =* 0.006). In the analysis of allele frequencies in both populations, the minor allele A showed a 1.51-fold increased risk of B-ALL (95% CI: 1.11–2.05; *p =* 0.007).

The second SNP was rs62571442 in miR-3689d2. The CT/CC genotype showed a 1.48-fold increased risk of B-ALL (95% CI: 1.02–2.15; *p =* 0.039) in the Spanish cohort. In the Slovenian cohort, a higher *p* value was observed (OR: 3.57; 95% CI: 1.57–8.12; *p =* 0.001). When both populations were analyzed together, they showed the same tendency (OR: 1.31; 95% CI: 1.06–1.60; *p =* 0.011). In the analysis of allele frequencies in both populations, the minor allele C showed a 1.31-fold increased risk of B-ALL (95% CI: 1.06–1.6; *p =* 0.012).

When we analyzed the Spanish cohort independently, other 13 SNPs in 13 miRNAs were significantly associated with B-ALL risk. None of these 13 SNPs were replicated in the Slovenian population (Supplementary Table 4). In the Slovenian cohort, other 11 SNPs in 10 miRNAs showed association with B-ALL risk and none of these 11 SNPs were replicated in the Spanish population (Supplementary Table 5). All the SNPs remained significantly associated with ALL risk after multivariate logistic regression to account for the possible confounding effect of sex. None of the SNPs reached statistical significance when FDR correction was applied.

### Bioinformatic analysis

#### miRNAs secondary structures prediction

We analyzed *in silico* the energy change (|ΔΔG|) and the secondary structures modifications of the SNPs associated with B-ALL risk in both populations (rs12402181 in miR-3117-3p and rs62571442 in miR-3689d2).

**Figure 1:**
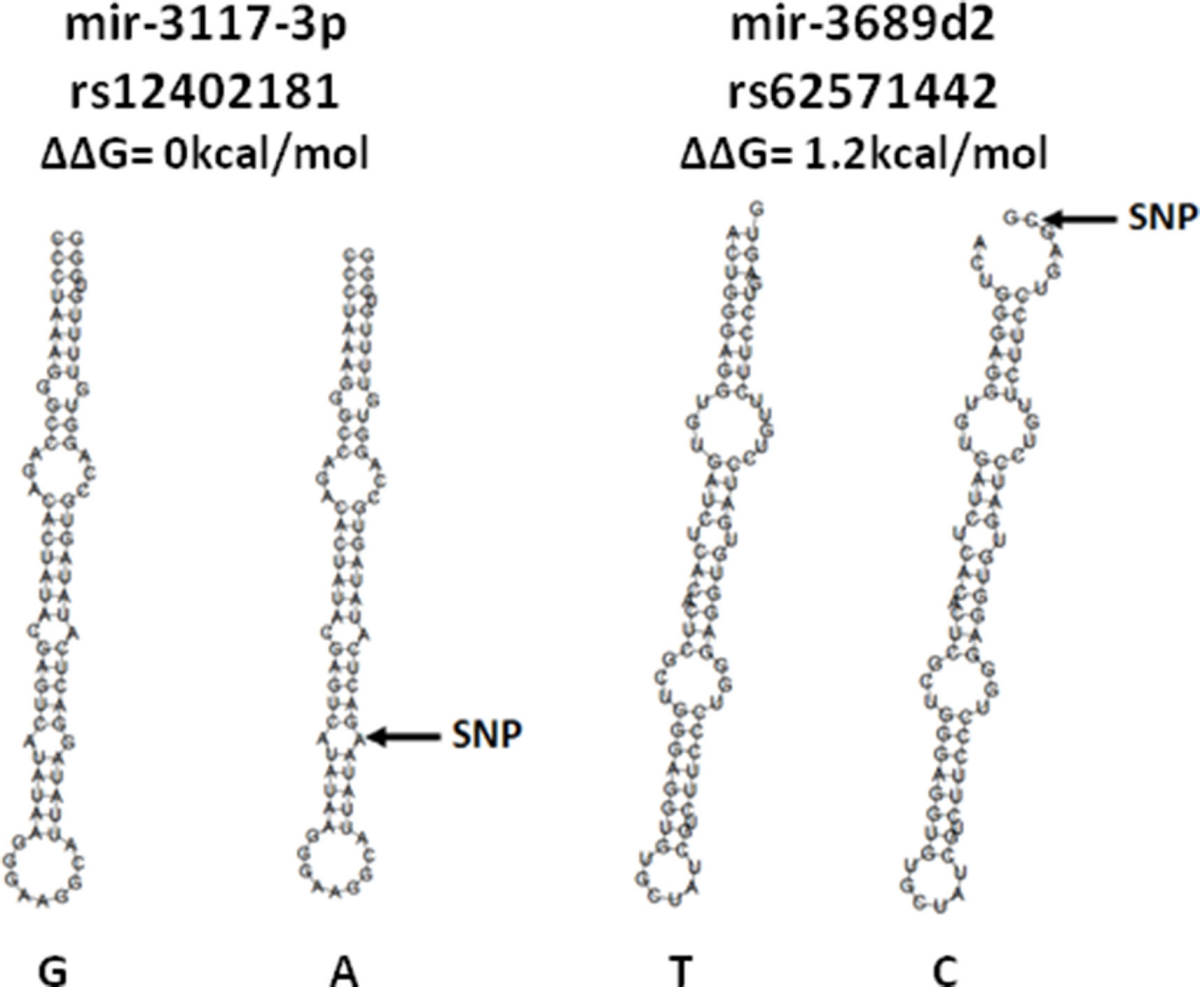
Secondary structures of the miRNAs with SNPs associated with B-ALL risk, predicted by the miRNA SNP tool

The SNP rs12402181 is located in the seed region of miR-3117-3p. The change from G to A allele did not show either an energy change or change in the secondary structure. On the other hand, rs62571442, located in the pre-miRNA of miR-3689d2, showed an energy change from -31.2 kcal/mol for the T allele to -30.0 kcal/mol for the risk allele C (1.2 kcal/mol). This SNP also produced an evident change in the secondary structure (Figure 1).

### Pathway analysis

In order to evaluate the pathways that could be affected by the miRNAs with the most significant SNPs, we first searched the target genes using miRWalk database and next, we performed a pathway enrichment analysis by using the ConsensusPathDB web tool.

Among the ten most significant pathways for miR-3117-3p, we found the mitogen-activated protein kinase (MAPK) signalling pathway over-represented (*p*-value of 4.9 × 10^-7^) (Supplementary Table 6). In this pathway, miR-3117-3p targeted up to 24 genes (Supplementary Table 7). Moreover, seven out of these ten enriched pathways were related to Ras signalling cascade, which is one of the MAPK pathways (Supplementary Table 6).

**Figure 2:**
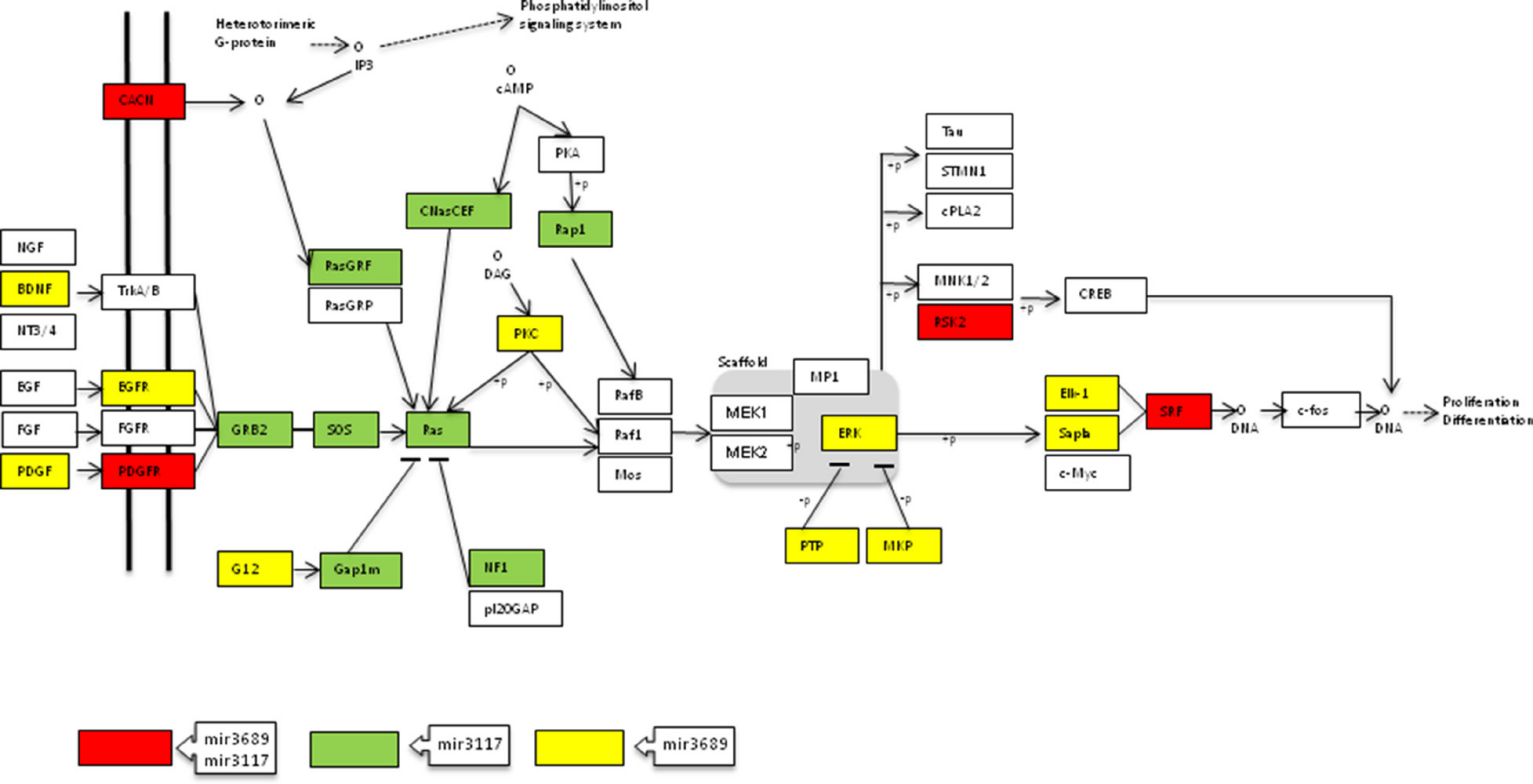
Genes of the MAPK signalling pathway targeted by miR-3117-3p and miR-3689d2 (adapted from KEGG database)

Regarding miR-3689d2, six out of ten most significant pathways were also related with Ras signalling, and this miRNA targeted up to 32 genes involved in MAPK signalling (Supplementary Tables 8 and 9). When we analyzed the putative target genes of both miRNAs together, the association for MAPK signalling pathway increased up to *p =* 5.75 × 10^-13^, with both miRNAs targeting up to 55 genes of the pathway. Moreover, eight out of the top ten pathways were related with Ras cascade (Figure 2 and Supplementary Table 10).

## DISCUSSION

In the current study, rs12402181 in miR-3117 and rs62571442 in miR-3689d2 showed statistically significant association with B-ALL risk in the two independent cohorts analyzed in this study, Spanish and Slovenian population. Our results point to a putative role of SNPs in miRNAs in B-ALL susceptibility.

The SNP rs12402181 in miR-3117-3p was associated with an increased risk of developing B-ALL under the additive model, A allele being the risk allele. This result was observed in the Spanish (*p =* 0.047) and the Slovenian population (*p =* 0.041). The association increased when both populations were analyzed together (*p =* 0.006). These results support the idea that rs12402181 A allele could represent a risk allele of low penetrance for B-ALL independently of the population studied. Rs12402181 is located in the seed region of miR-3117-3p, a miRNA expressed in B-ALL [20], therefore, the change of the G allele for the A allele could affect the accurate recognition of its target mRNA sequences. The loss of binding could increase the expression of its target genes. Among the target genes of miR-3117-3p, *in silico* analysis showed that genes of MAPK signalling pathway are over-represented (Figure 2), including mainly genes of the MAPK/ERK family or classical pathway [31–33]. Interestingly, the genes predicted to be targeted by miR-3117-3p are in the first steps of the cascade (*CACNG1, CACNG8*, *PDGFR*, *GRB2*, *SOS1* and *RAS),* which in turn could produce the deregulation of the following steps. Aberrant expression of this pathway is a major and highly prevalent oncogenic event in many human cancers [34], including childhood ALL [35, 36]. In summary, failed recognition between miR-3117-3p and its targets due to the change of the rs12402181 G allele to the A allele in the seed region could contribute to leukemogenesis by leading to an aberrant activation of the RAS-MAPK pathway.

The second SNP, rs62571442 in miR-3689d2, was associated with an increased risk of B-ALL under the additive model, C allele being the risk allele. This result was observed in the Spanish cohort (*p =* 0.039), as well as in the Slovenian population (*p =* 0.001), indicating that this SNP could be a general marker for B-ALL. The C > T change in this SNP, located in the pre-miRNA sequence, modified the secondary structure and induced a positive energy change of 1.2 kcal/mol for the C risk allele. The hairpin structure changed from stable (U:A) to unstable status (C:A). When the SNP decreases the stability, the production of mature miRNA is reduced, which in turn may increase target gene expression [37]. In the pathway analysis of miR-3689d2, six out of the top ten enriched pathways were again related to the aforementioned Ras signalling. Therefore, a decreased expression of this miRNA could increase the expression of RAS-MAPK pathway genes.

When pathways analysis for miR-3117-3p and miR-3689d2 were performed together, eight out of the ten first pathways were related to RAS-MAPK signalling, and the association obtained was higher. Therefore, both miRNAs could contribute to the activation of this pathway in a synergistic way: on the one hand, because of the loss of target recognition due to the A allele of the SNP rs12402181 in the seed region of miR-3117-3p, and on the other hand, because of the reduction in mature levels of miR-3689d2 due to the risk allele C of rs62571442 in the hairpin structure. These results were found in two different populations, therefore, they could be considered as general markers of B-ALL susceptibility. It would be interesting to study these associations in other populations.

This study has some limitations that might be addressed, such as the relatively high failure rate in genotyping technique (63.4%). However, this high chance of failure was accepted from the beginning of the study, because despite the predicted low score for genotyping, no other design option to amplify the polymorphisms in question was possible. Moreover, these results did not reach a significant *p*-value when FDR correction was applied, but we would like to emphasize that both SNPs were significant in two independent cohorts. Another possible weakness is the known inaccuracy of the miRNA target prediction algorithms of the databases used [38, 39].

In conclusion, rs12402181 in miR-3117-3p and rs62571442 in miR-3689d2 could be involved in B-ALL susceptibility through their effect on the regulation of MAPK signalling-related pathways.

## MATERIALS AND METHODS

### Study participants

A total of 310 Caucasian children diagnosed with B-ALL and 434 unrelated healthy controls were included in this study (Table 1). The Spanish cohort consisted of 231 children diagnosed with B-ALL between 2000 and 2011 in the Pediatric Oncology Units of four Spanish hospitals (University Hospital Cruces, University Hospital Donostia, University Hospital La Paz and University Hospital Miguel Servet) and 338 unrelated healthy individuals. The Slovenian cohort consisted of 79 Caucasian children diagnosed with B-ALL between 1993 and 2009, at the Department of Hematology and Oncology of the University Children’s Hospital Ljubljana and 96 unrelated healthy individuals.

Data were collected objectively, blinded to genotypes, from the patients’ medical files. Sex and age data were systematically recorded from the clinical records (Table 1). Informed consent was obtained from all participants, or from their parents prior to sample collection. The study was approved by the ethics committees (PI2014039 and 62/07/03) and was carried out according to the Declaration of Helsinki.

### Selection of genes and polymorphisms

We selected all the SNPs in pre-miRNAs with a MAF > 0.01 in European/Caucasian populations described in the databases until May 2014. We decided to include all miRNAs due to the fact that they can regulate a wide range of genes that are not completely defined. Therefore, any miRNA could be implicated in the regulation of genes affecting B-ALL risk. Of a total of 1910 SNPs in 969 miRNAs found at the moment of the study, we included all the SNPs with a MAF > 0.01, a total of 213 SNPs in 206 pre-miRNAs (Supplementary Table 1). The SNP selection was performed using miRNA SNiPer (www.integratomics-time.com/miRNA-SNiPer/), NCBI (http://www.ncbi.nlm.nih.gov/snp/) and literature review.

### Genotype analyses

Genomic DNA was extracted from remission peripheral blood or bone marrow (with < 5% blast cells) as previously described [40]. DNA was quantified using PicoGreen (Invitrogen Corp., Carlsbad, CA).

For each sample, 400 ng of DNA were genotyped using the GoldenGate Genotyping Assay with Veracode technology according to the published Illumina protocol. Data were analyzed with GenomeStudio software for genotype clustering and calling. Duplicate samples and CEPH trios (Coriell Cell Repository, Camden, NJ) were genotyped across the plates. SNPs showing Mendelian allele-transmission errors or showing discordant genotypes were excluded from the analysis.

In order to validate the genotyping, 175 samples and 15 SNPs were genotyped using TaqMan OpenArray Genotyping technology according to the published Applied Biosystems protocol and analyzed using Taqman Genotyper software (Applied Biosystems, Carlsbad, CA).

### Statistical analysis

To identify any deviation from Hardy-Weinberg equilibrium (HWE) for the healthy controls, a χ^2^ test was used. The association between genetic polymorphisms in cases and controls was also evaluated using the χ^2^ or Fisher’s exact test. The effect sizes of the associations were estimated by the odds ratio from univariate logistic regression and multivariate logistic regression to account for the possible confounding effect of sex. The most significant test among codominant (major allele homozygotes *vs.* heterozygotes and major allele homozygotes *vs.* minor allele homozygotes), dominant (major allele homozygotes *vs*. heterozygotes + minor allele homozygotes), recessive (major allele homozygotes + heterozygotes *vs*. minor allele homozygotes), and additive (doses-dependent effect: major allele homozygotes *vs*. heterozygotes *vs*. minor allele homozygotes) genetic models was selected. The results were adjusted for multiple comparisons by the False Discovery Rate (FDR) [41]. In all cases the significance level was set at 5%. Analyses were performed using R v2.11 software.

### Bioinformatic analysis

#### miRNAs secondary structures prediction

The RNAfold web tool (http://rna.tbi.univie.ac.at) was used to calculate the minimum free energy (MFE) secondary structures and to predict the most stable secondary structures of the miRNAs with statistically significant SNPs.

#### Gene targets selection and pathways analysis

MiRWalk [32] (http://zmf.umm.uni-heidelberg.de/apps/zmf/mirwalk2/) database was used to select miRNA targets. Targets predicted by at least 6 different algorithms provided by miRWalk were selected. Enriched pathway analyses of putative target genes were determined with ConsensusPath database (CPdB) (http://consensuspathdb.org/) [31] using the over-representation analysis module. Gene lists were analyzed against the default collection of KEGG [33], Reactome [42] and BioCarta (http://cgap.nci.nih.gov/Pathways/BioCarta_Pathways) pathway databases. A conservative *p*-value cutoff (0.0001) was used.

## SUPPLEMENTARY MATERIALS TABLES











## Abbreviations

(ALL): Acute lymphoblastic leukemia
(miRNAs): microRNAs
(MAF): minor allele frequency
(GWAS): genome-wide association studies
(UTR): untranslated region
(BCR): B-cell receptor
(HWE): Hardy-Weinberg equilibrium
(FDR): False Discovery Rate
(MFE): minimum free energy
(MAPK): mitogen-activated protein kinase
