# Supplementary material for: Involvement of SNPs in miR-3117 and miR-3689d2 in childhood acute lymphoblastic leukemia risk
Source: Oncotarget. 2018 May 1;9(33):22907–14. doi: 10.18632/oncotarget.25144 (PMC5955428; doi:10.18632/oncotarget.25144)
Supplement: Supplementary file 1 [file oncotarget-09-22907-s001.pdf]

Involvement of SNPs in miR-3117 and miR-3689d2 in childhood acute lymphoblastic leukemia risk

SUPPLEMENTARY MATERIALS

Supplementary Table 1: SNPs selected for the study. See_Supplementary_Table_1

Supplementary Table 2: Comparison between goldengate array and taqman open array methods for genotyping

SNP	Genotype	GoldenGate	Taqman	Concordance (%)
**rs10061133**	AA	156	156	100%
	AG	18	18	
	GG	1	1	
	NA	0	0	
**rs10173558**	TT	140	134	100%
	CT	34	32	
	CC	1	1	
	NA	0	8	
**rs10505168**	AA	73	72	100%
	AG	83	81	
	GG	18	18	
	NA	1	4	
**rs11156654**	TT	105	104	100%
	AT	55	50	
	AA	14	12	
	NA	1	9	
**rs11259096**	TT	151	151	98.9%
	CT	22	23	
	CC	2	0	
	NA	0	1	
**rs12803915**	GG	130	128	100%
	AG	43	40	
	AA	2	2	
	NA	0	5	
**rs17091403**	CC	152	151	99.4%
	CT	21	22	
	TT	2	2	
	NA	0	0	
**rs2043556**	AA	98	89	100%
	AG	62	58	
	GG	12	13	
	NA	3	15	
**rs2289030**	CC	155	154	98.9%
	CG	18	20	
	GG	1	1	
	NA	1	0	
**rs34115976**	CC	115	115	100%
	CG	52	53	
	GG	6	5	
	NA	2	2	
**rs41291179**	AA	158	156	100%
	AT	15	15	
	TT	2	2	
	NA	0	2	
**rs4909237**	CC	120	118	100%
	CT	44	44	
	TT	10	11	
	NA	1	2	
**rs4919510**	CC	106	105	100%
	CG	60	61	
	GG	7	8	
	NA	2	1	
**rs56103835**	TT	110	110	100%
	CT	55	54	
	CC	10	8	
	NA	0	3	
**rs58834075**	CC	166	164	100%
	CT	7	8	
	TT	1	1	
	NA	1	2	

Supplementary Table 3: Polymorphisms in miRNAs associated with B-ALL risk in the spanish and slovenian population. See_Supplementary_Table_3

Supplementary Table 4: Polymorphisms in miRNAs associated with B-ALL risk in the spanish cohort. See_Supplementary_Table_4

Supplementary Table 5: Polymorphisms in miRNAs associated with B-ALL risk in the slovenian cohort. See_Supplementary_Table_5

Supplementary Table 6: Enriched pathways for miR-3117-3p predicted target genes

Pathway name	Set size	Candidates	*p*-value	*q*-value	Pathway source
**MAPK signaling pathway - Homo sapiens (human)**	257	24 (9.3%)	4.94e-07	0.000322	**KEGG**
**Ras signaling pathway - Homo sapiens (human)**	228	21 (9.2%)	3.24e-06	0.00106	**KEGG**
**Choline metabolism in cancer - Homo sapiens (human)**	101	13 (12.9%)	7.18e-06	0.00149	**KEGG**
**PDGF signaling pathway**	27	7 (25.9%)	9.14e-06	0.00149	**BioCarta**
**Renal cell carcinoma - Homo sapiens (human)**	66	10 (15.2%)	1.95e-05	0.00254	**KEGG**
**FoxO signaling pathway - Homo sapiens (human)**	134	14 (10.4%)	3.61e-05	0.00317	**KEGG**
**ErbB signaling pathway - Homo sapiens (human)**	87	11 (12.6%)	4.32e-05	0.00317	**KEGG**
**Signaling by EGFR in Cancer**	15	5 (33.3%)	4.87e-05	0.00317	**Reactome**
**Constitutive Signaling by Ligand-Responsive EGFR Cancer Variants**	15	5 (33.3%)	4.87e-05	0.00317	**Reactome**
**Signaling by Ligand-Responsive EGFR Variants in Cancer**	15	5 (33.3%)	4.87e-05	0.00317	**Reactome**
**Diseases of signal transduction**	180	16 (8.9%)	7.45e-05	0.00442	**Reactome**

Supplementary Table 7: Genes of MAPK signaling pathway targeted by miR-3117-3p

Entrez-gene ID	*Entrez-gene name*
**3845**	KRAS: Kirsten rat sarcoma viral oncogene homolog
**5156**	PDGFRA: platelet-derived growth factor receptor, alpha polypeptide
**6197**	RPS6KA3: ribosomal protein S6 kinase, 90kDa, polypeptide 3
**51776**	ZAK: sterile alpha motif and leucine zipper containing kinase AZK
**6722**	SRF: serum response factor (c-fos serum response element-binding transcription factor)
**2317**	FLNB: filamin B, beta
**23162**	MAPK8IP3: mitogen-activated protein kinase 8 interacting protein 3
**6789**	STK4: serine/threonine kinase 4
**4763**	NF1: neurofibromin 1
**6416**	MAP2K4: mitogen-activated protein kinase kinase 4
**8913**	CACNA1G: calcium channel, voltage-dependent, T type, alpha 1G subunit
**786**	CACNG1: calcium channel, voltage-dependent, gamma subunit 1
**5908**	RAP1B: RAP1B, member of RAS oncogene family
**10000**	AKT3: v-akt murine thymoma viral oncogene homolog 3
**5922**	RASA2: RAS p21 protein activator 2
**5923**	RASGRF1: Ras protein-specific guanine nucleotide-releasing factor 1
**5924**	RASGRF2: Ras protein-specific guanine nucleotide-releasing factor 2
**2885**	GRB2: growth factor receptor-bound protein 2
**1386**	ATF2: activating transcription factor 2
**7046**	TGFBR1: transforming growth factor, beta receptor 1
**59283**	CACNG8: calcium channel, voltage-dependent, gamma subunit 8
**9693**	RAPGEF2: Rap guanine nucleotide exchange factor (GEF) 2
**5599**	MAPK8: mitogen-activated protein kinase 8
**6654**	SOS1: son of sevenless homolog 1 (Drosophila)

Supplementary Table 8: The top ten enriched pathways for miR-3689d2 predicted target genes

**Pathway name**	**Set size**	**Candidates**	***p*-value**	***q*-value**	**Pathway source**
**Axon guidance**	459	59 (12.9%)	4.69e-13	4.24e-10	**Reactome**
**Developmental Biology**	586	68 (11.6%)	9.47e-13	4.28e-10	**Reactome**
**NGF signalling via TRKA from the plasma membrane**	310	43 (13.9%)	7.24e-11	1.84e-08	**Reactome**
**Signalling by NGF**	386	49 (12.7%)	8.14e-11	1.84e-08	**Reactome**
**Signaling by PDGF**	301	41 (13.6%)	3.61e-10	6.52e-08	**Reactome**
**Signaling by EGFR**	292	40 (13.7%)	5.03e-10	7.57e-08	**Reactome**
**DAP12 interactions**	298	40 (13.4%)	9.29e-10	1.2e-07	**Reactome**
**Downstream signal transduction**	279	38 (13.6%)	1.64e-09	1.25e-07	**Reactome**
**Downstream signaling of activated FGFR2**	267	37 (13.9%)	1.66e-09	1.25e-07	**Reactome**
**Downstream signaling of activated FGFR1**	267	37 (13.9%)	1.66e-09	1.25e-07	**Reactome**

Supplementary Table 9: Genes of MAPK signaling pathway targeted by miR-3689d2

entrez-gene ID	*entrez-gene name*
**5154**	PDGFA: platelet-derived growth factor alpha polypeptide
**5155**	PDGFB: platelet-derived growth factor beta polypeptide
**5159**	PDGFRB: platelet-derived growth factor receptor, beta polypeptide
**4137**	MAPT: microtubule-associated protein tau
**9479**	MAPK8IP1: mitogen-activated protein kinase 8 interacting protein 1
**6195**	RPS6KA1: ribosomal protein S6 kinase, 90kDa, polypeptide 1
**6722**	SRF: serum response factor (c-fos serum response element-binding transcription factor)
**208**	AKT2: v-akt murine thymoma viral oncogene homolog 2
**627**	BDNF: brain-derived neurotrophic factor
**5778**	PTPN7: protein tyrosine phosphatase, non-receptor type 7
**51347**	TAOK3: TAO kinase 3
**5494**	PPM1A: protein phosphatase, Mg2+/Mn2 + dependent, 1A
**2768**	GNA12: guanine nucleotide binding protein (G protein) alpha 12
**2250**	FGF5: fibroblast growth factor 5
**10454**	TAB1: TGF-beta activated kinase 1/MAP3K7 binding protein 1
**782**	CACNB1: calcium channel, voltage-dependent, beta 1 subunit
**10000**	AKT3: v-akt murine thymoma viral oncogene homolog 3
**1850**	DUSP8: dual specificity phosphatase 8
**1852**	DUSP9: dual specificity phosphatase 9
**57551**	TAOK1: TAO kinase 1
**5609**	MAP2K7: mitogen-activated protein kinase kinase 7
**5532**	PPP3CB: protein phosphatase 3, catalytic subunit, beta isozyme
**1956**	EGFR: epidermal growth factor receptor
**5058**	PAK1: p21 protein (Cdc42/Rac)-activated kinase 1
**5578**	PRKCA: protein kinase C, alpha
**5579**	PRKCB: protein kinase C, beta
**2002**	ELK1: ELK1, member of ETS oncogene family
**27092**	CACNG4: calcium channel, voltage-dependent, gamma subunit 4
**2005**	ELK4: ELK4, ETS-domain protein (SRF accessory protein 1)
**5594**	MAPK1: mitogen-activated protein kinase 1
**5595**	MAPK3: mitogen-activated protein kinase 3
**3554**	IL1R1: interleukin 1 receptor, type I

Supplementary Table 10: Top ten enriched pathways for miR-3117-3p and miR-3689d2 predicted target genes

Pathway name	Set size	Candidates contained	*p*-value	*q*-value	Pathway source
**Axon guidance**	459	83 (18.1%)	2.77e-15	3.13e-12	**Reactome**
**Developmental Biology**	586	96 (16.4%)	1.09e-14	6.16e-12	**Reactome**
**NGF signalling via TRKA from the plasma membrane**	310	62 (20.0%)	1.1e-13	3.86e-11	**Reactome**
**Signalling by NGF**	386	71 (18.4%)	1.37e-13	3.86e-11	**Reactome**
**MAPK signaling pathway - Homo sapiens (human)**	257	54 (21.0%)	5.75e-13	1.3e-10	**KEGG**
**Signaling by EGFR**	292	58 (19.9%)	9.62e-13	1.65e-10	**Reactome**
**Signaling by PDGF**	301	59 (19.6%)	1.1e-12	1.65e-10	**Reactome**
**Signaling by FGFR3**	270	55 (20.4%)	1.32e-12	1.65e-10	**Reactome**
**Signaling by FGFR4**	270	55 (20.4%)	1.32e-12	1.65e-10	**Reactome**
**Signaling by FGFR1**	271	55 (20.3%)	1.54e-12	1.74e-10	**Reactome**
