# Supplementary material for: Involvement of SNPs in miR-3117 and miR-3689d2 in childhood acute lymphoblastic leukemia risk
Source: Oncotarget. 2018 May 1;9(33):22907–14. doi: 10.18632/oncotarget.25144 (PMC5955428; doi:10.18632/oncotarget.25144)
Supplement: Supplementary file 2 [file oncotarget-09-22907-s002.docx]

| **Supplementary Table 1: SNPs selected for the study** | | | | | | |
| --- | --- | --- | --- | --- | --- | --- |
| **N** | **SNP** | **Gene** | **% Genotyping** | **MAF** | **Alleles** | **Exclusion criteria** |
| 1 | **rs10061133** | hsa-mir-449b | 100.0 | 0.079 | A:G |  |
| 2 | **rs10173558** | mir-1302-4 | 100.0 | 0.123 | T:C |  |
| 3 | **rs10406069** | hsa-mir-5196 | 99.3 | 0.209 | G:A |  |
| 4 | **rs10422347** | hsa-mir-4745 | 98.2 | 0.082 | C:T |  |
| 5 | **rs10461441** | hsa-mir-548ae-2 | 0.0 | 0.0 | T:T | Genotyping failure |
| 6 | **rs10505168** | hsa-mir-2053 | 99.4 | 0.307 | A:G |  |
| 7 | **rs1055070** | hsa-mir-4700 | 99.7 | 0.059 | T:G |  |
| 8 | **rs1077020** | hsa-mir-943 | 0.0 | 0.0 | T:T | Genotyping failure |
| 9 | **rs10878362** | hsa-mir-6074 | 0.0 | 0.0 | T:T | Genotyping failure |
| 10 | **rs10934682** | hsa-mir-544b | 100.0 | 0.159 | T:G |  |
| 11 | **rs11014002** | hsa-mir-603 | 0.0 | 0.0 | T:T | Genotyping failure |
| 12 | **rs11032942** | hsa-mir-1343 | 0.0 | 0.0 | T:T | Genotyping failure |
| 13 | **rs11156654** | mir-624 | 99.3 | 0.248 | T:A |  |
| 14 | **rs11237828** | hsa-mir-5579 | 0.0 | 0.0 | T:T | Genotyping failure |
| 15 | **rs11259096** | hsa-mir-1265 | 99.9 | 0.056 | T:C |  |
| 16 | **rs11614913** | hsa-mir-196a-2 | 99.7 | 0.349 | C:T |  |
| 17 | **rs11651671** | hsa-mir-548at | 0.0 | 0.0 | T:T | Genotyping failure |
| 18 | **rs11713052** | hsa-mir-5092 | 100.0 | 0.038 | C:G |  |
| 19 | **rs11714172** | hsa-mir-4792 | 99.2 | 0.363 | T:G |  |
| 20 | **rs11907020** | hsa-mir-3192 | 99.9 | 0.02 | T:C |  |
| 21 | **rs11983381** | hsa-mir-4653 | 97.1 | 0.188 | A:G |  |
| 22 | **rs12197631** | hsa-mir-548a-1 | 0.0 | 0.0 | T:T | Genotyping failure |
| 23 | **rs12355840** | hsa-mir-202 | 85.7 | 0.185 | T:C | No HWE Slovenia  No HWE total |
| 24 | **rs12402181** | hsa-mir-3117 | 100.0 | 0.135 | G:A |  |
| 25 | **rs12451747** | hsa-mir-1269b | 0.0 | 0.0 | T:T | Genotyping failure |
| 26 | **rs12456845** | hsa-mir-4744 | 99.9 | 0.036 | T:C |  |
| 27 | **rs12473206** | hsa-mir-4433 | 0.0 | 0.0 | T:T | Genotyping failure |
| 28 | **rs12512664** | hsa-mir-4274 | 99.2 | 0.471 | A:G |  |
| 29 | **rs12523324** | hsa-mir-4277 | 0.0 | 0.0 | T:T | Genotyping failure |
| 30 | **rs12780876** | hsa-mir-4293 | 99.4 | 0.289 | T:A |  |
| 31 | **rs12803915** | hsa-mir-612 | 99.3 | 0.161 | G:A |  |
| 32 | **rs12879262** | hsa-mir-4309 | 99.7 | 0.145 | G:C |  |
| 33 | **rs12894467** | hsa-mir-300 | 99.7 | 0.385 | C:T |  |
| 34 | **rs13186787** | hsa-mir-1294 | 0.0 | 0.0 | T:T | Genotyping failure |
| 35 | **rs13299349** | hsa-mir-3152 | 85.5 | 0.348 | G:A |  |
| 36 | **rs1414273** | hsa-mir-548ac | 0.0 | 0.0 | T:T | Genotyping failure |
| 37 | **rs1439619** | hsa-mir-3175 | 86.1 | 0.498 | C:A |  |
| 38 | **rs1572687** | hsa-mir-5007 | 99.4 | 0.45 | C:T |  |
| 39 | **rs1683709** | hsa-mir-3612 | 100.0 | 0.199 | C:T |  |
| 40 | **rs17022749** | hsa-mir-5700 | 0.0 | 0.0 | T:T | Genotyping failure |
| 41 | **rs17091403** | hsa-mir-2110 | 99.9 | 0.089 | C:T |  |
| 42 | **rs17111728** | hsa-mir-4422 | 100.0 | 0.068 | T:C |  |
| 43 | **rs174561** | hsa-mir-1908 | 57.0 | 0.254 | T:C | Genotyping failure |
| 44 | **rs17737028** | hsa-mir-3143 | 100.0 | 0.008 | A:G |  |
| 45 | **rs17759989** | hsa-mir-633 | 99.9 | 0.017 | A:G |  |
| 46 | **rs17797090** | hsa-mir-3652 | 99.9 | 0.109 | G:A |  |
| 47 | **rs17885221** | hsa-mir-4733 | 99.7 | 0.057 | C:T |  |
| 48 | **rs2042253** | hsa-mir-5197 | 99.6 | 0.22 | A:G |  |
| 49 | **rs2043556** | hsa-mir-605 | 99.0 | 0.218 | A:G |  |
| 50 | **rs2060455** | hsa-mir-4511 | 0.0 | 0.0 | T:T | Genotyping failure |
| 51 | **rs2070960** | hsa-mir-3620 | 99.2 | 0.061 | C:T |  |
| 52 | **rs2114358** | hsa-mir-1206 | 99.2 | 0.433 | T:C |  |
| 53 | **rs215383** | hsa-mir-4494 | 99.2 | 0.178 | G:A |  |
| 54 | **rs2241347** | hsa-mir-3130-1 | 0.0 | 0.0 | T:T | Genotyping failure |
| 55 | **rs2273626** | hsa-mir-4707 | 89.0 | 0.471 | A:C |  |
| 56 | **rs2289030** | hsa-mir-492 | 99.7 | 0.068 | C:G |  |
| 57 | **rs2291418** | hsa-mir-1229 | 100.0 | 0.03 | C:T |  |
| 58 | **rs2292181** | hsa-mir-564 | 99.7 | 0.042 | G:C |  |
| 59 | **rs2292832** | hsa-mir-149 | 0.0 | 0.0 | T:T | Genotyping failure |
| 60 | **rs2368392** | hsa-mir-604 | 99.4 | 0.248 | C:T | No HWE Spain |
| 61 | **rs243080** | hsa-mir-4432 | 98.3 | 0.43 | C:T |  |
| 62 | **rs257095** | hsa-mir-4636 | 99.7 | 0.154 | A:G |  |
| 63 | **rs2648841** | hsa-mir-1208 | 98.9 | 0.123 | C:A |  |
| 64 | **rs2663345** | hsa-mir-3183 | 0.0 | 0.0 | T:T | Genotyping failure |
| 65 | **rs266435** | hsa-mir-4804 | 99.3 | 0.136 | C:G |  |
| 66 | **rs2682818** | hsa-mir-618 | 99.3 | 0.131 | C:A |  |
| 67 | **rs28477407** | hsa-mir-4308 | 100.0 | 0.088 | C:T |  |
| 68 | **rs28645567** | hsa-mir-378d-1 | 99.7 | 0.015 | G:A |  |
| 69 | **rs28655823** | hsa-mir-4472-1 | 89.6 | 0.114 | G:C |  |
| 70 | **rs28664200** | hsa-mir-1255a | 82.3 | 0.247 | T:C |  |
| 71 | **rs2910164** | hsa-mir-146a | 99.9 | 0.249 | G:C |  |
| 72 | **rs2967897** | hsa-mir-5695 | 100.0 | 0.0 | G:G | Monomorphic |
| 73 | **rs3112399** | hsa-mir-4803 | 99.3 | 0.447 | T:A | No HWE Spain  No HWE total |
| 74 | **rs34115976** | hsa-mir-577 | 98.9 | 0.196 | C:G | No HWE Spain |
| 75 | **rs35196866** | hsa-mir-4669 | 0.0 | 0.0 | T:T | Genotyping failure |
| 76 | **rs356125** | hsa-mir-2278 | 99.9 | 0.052 | G:A |  |
| 77 | **rs35613341** | hsa-mir-5189 | 99.6 | 0.339 | C:G | No HWE Spain  No HWE total |
| 78 | **rs35650931** | hsa-mir-6076 | 99.9 | 0.094 | G:C |  |
| 79 | **rs35770269** | hsa-mir-449c | 99.6 | 0.345 | A:T |  |
| 80 | **rs35854553** | hsa-mir-3166 | 86.5 | 0.068 | A:T |  |
| 81 | **rs367805** | hsa-mir-3936 | 99.0 | 0.309 | G:A |  |
| 82 | **rs3734050** | hsa-mir-6499 | 99.9 | 0.053 | C:T |  |
| 83 | **rs3746444** | hsa-mir-499a | 99.3 | 0.205 | T:C |  |
| 84 | **rs3823658** | hsa-mir-5090 | 99.7 | 0.141 | G:A |  |
| 85 | **rs4112253** | hsa-mir-4751 | 99.9 | 0.365 | C:G |  |
| 86 | **rs41274239** | hsa-mir-96 | 99.7 | 0.001 | A:G |  |
| 87 | **rs41274312** | hsa-mir-187 | 99.7 | 0.006 | G:A |  |
| 88 | **rs41286570** | hsa-mir-154 | 100.0 | 0.0 | G:G | Monomorphic |
| 89 | **rs41291179** | hsa-mir-216a | 100.0 | 0.056 | A:T |  |
| 90 | **rs41292412** | hsa-mir-122 | 99.9 | 0.002 | C:T |  |
| 91 | **rs4285314** | hsa-mir-3135b | 0.0 | 0.0 | T:T | Genotyping failure |
| 92 | **rs4414449** | hsa-mir-548ap | 81.3 | 0.376 | T:C |  |
| 93 | **rs45530340** | hsa-mir-6084 | 99.9 | 0.0 | C:C | Monomorphic |
| 94 | **rs4577031** | hsa-mir-548ap | 99.6 | 0.366 | A:T |  |
| 95 | **rs4674470** | hsa-mir-4268 | 99.7 | 0.214 | T:C |  |
| 96 | **rs4809383** | hsa-mir-941-1 | 86.4 | 0.117 | C:T | No HWE Spain |
| 97 | **rs4822739** | hsa-mir-548j | 99.9 | 0.063 | C:G |  |
| 98 | **rs487571** | hsa-mir-5680 | 0.0 | 0.0 | T:T | Genotyping failure |
| 99 | **rs4909237** | hsa-mir-595 | 99.6 | 0.164 | C:T |  |
| 100 | **rs4919510** | hsa-mir-608 | 99.4 | 0.198 | C:G |  |
| 101 | **rs515924** | hsa-mir-548al | 99.3 | 0.1 | A:G |  |
| 102 | **rs521188** | hsa-mir-3671 | 100.0 | 0.035 | A:G |  |
| 103 | **rs56088671** | hsa-mir-4424 | 0.0 | 0.0 | T:T | Genotyping failure |
| 104 | **rs56103835** | hsa-mir-323b | 100.0 | 0.179 | T:C |  |
| 105 | **rs56195815** | hsa-mir-548aw | 0.0 | 0.0 | T:T | Genotyping failure |
| 106 | **rs56292801** | hsa-mir-5189 | 89.1 | 0.299 | G:A | Ho HWE Spain  No HWE total |
| 107 | **rs57111412** | hsa-mir-1283-1 | 0.0 | 0.0 | T:T | Genotyping failure |
| 108 | **rs58450758** | hsa-mir-559 | 0.0 | 0.0 | T:T | Genotyping failure |
| 109 | **rs58834075** | hsa-mir-656 | 99.9 | 0.02 | C:T |  |
| 110 | **rs5965660** | hsa-mir-888 | 99.9 | 0.157 | T:G | No HWE Spain |
|  |  |  |  |  |  | No HWE Slovenia |
| 111 | **rs5997893** | hsa-mir-3928 | 99.3 | 0.319 | G:A |  |
| 112 | **rs60308683** | hsa-mir-4762 | 0.0 | 0.0 | T:T | Genotyping failure |
| 113 | **rs6062431** | hsa-mir-4326 | 98.6 | 0.328 | G:C |  |
| 114 | **rs60871950** | hsa-mir-4467 | 98.7 | 0.475 | G:A |  |
| 115 | **rs61388742** | hsa-mir-596 | 99.6 | 0.094 | T:C |  |
| 116 | **rs61938575** | hsa-mir-3922 | 85.9 | 0.285 | G:A |  |
| 117 | **rs61992671** | hsa-mir-412 | 99.6 | 0.493 | G:A |  |
| 118 | **rs62154973** | hsa-mir-4772 | 99.2 | 0.101 | C:T |  |
| 119 | **rs62376935** | hsa-mir-585 | 99.4 | 0.066 | C:T | No HWE Slovenia  No HWE total |
| 120 | **rs641071** | hsa-mir-4482 | 0.0 | 0.0 | T:T | Genotyping failure |
| 121 | **rs6430498** | hsa-mir-3679 | 99.0 | 0.326 | G:A |  |
| 122 | **rs6505162** | hsa-mir-423 | 0.0 | 0.0 | T:T | Genotyping failure |
| 123 | **rs6513496** | hsa-mir-646 | 99.6 | 0.197 | T:C |  |
| 124 | **rs66507245** | hsa-mir-4731 | 0.0 | 0.0 | T:T | Genotyping failure |
| 125 | **rs66683138** | hsa-mir-3622a | 0.0 | 0.0 | T:T | Genotyping failure |
| 126 | **rs67042258** | hsa-mir-6128 | 99.4 | 0.26 | G:A |  |
| 127 | **rs670637** | hsa-mir-3167 | 94.7 | 0.0 | T:T | Monomorphic |
| 128 | **rs67182313** | hsa-mir-4642 | 99.4 | 0.182 | A:G |  |
| 129 | **rs6726779** | hsa-mir-4431 | 99.4 | 0.377 | T:C |  |
| 130 | **rs67339585** | MIR3910-1, MIR3910-2 | 0.0 | 0.0 | T:T | Genotyping failure |
| 131 | **rs6787734** | hsa-mir-3135a | 0.0 | 0.0 | T:T | Genotyping failure |
| 132 | **rs67976778** | hsa-mir-4305 | 0.0 | 0.0 | T:T | Genotyping failure |
| 133 | **rs68035463** | hsa-mir-3144 | 99.4 | 0.223 | C:A |  |
| 134 | **rs6841938** | hsa-mir-1255b-1 | 0.0 | 0.0 | T:T | Genotyping failure |
| 135 | **rs6977967** | hsa-mir-3683 | 99.9 | 0.193 | A:G |  |
| 136 | **rs6997249** | hsa-mir-3686 | 0.0 | 0.0 | T:T | Genotyping failure |
| 137 | **rs701213** | hsa-mir-4427 | 0.0 | 0.0 | T:T | Genotyping failure |
| 138 | **rs702742** | hsa-mir-378h | 99.9 | 0.101 | A:G |  |
| 139 | **rs7070684** | hsa-mir-548aj-2 | 0.0 | 0.0 | T:T | Genotyping failure |
| 140 | **rs71363366** | hsa-mir-1283-2 | 99.2 | 0.038 | C:G |  |
| 141 | **rs7205289** | hsa-mir-140 | 87.5 | 0.0 | C:C | Monomorphic |
| 142 | **rs7207008** | hsa-mir-2117 | 99.3 | 0.468 | T:A |  |
| 143 | **rs7227168** | hsa-mir-4741 | 99.3 | 0.116 | C:T |  |
| 144 | **rs7247237** | hsa-mir-3188 | 99.2 | 0.291 | C:T |  |
| 145 | **rs72502717** | hsa-mir-3689f | 0.0 | 0.0 | T:T | Genotyping failure |
| 146 | **rs72631816** | hsa-mir-105-2 | 100.0 | 0.0 | T:T | Monomorphic |
| 147 | **rs72631825** | hsa-mir-222 | 100.0 | 0.0 | G:G | Monomorphic |
| 148 | **rs72631826** | hsa-mir-16-1 | 99.9 | 0.0 | T:T | Monomorphic |
| 149 | **rs72631827** | hsa-mir-106b | 99.9 | 0.0 | G:G | Monomorphic |
| 150 | **rs72631831** | hsa-mir-323b | 100.0 | 0.0 | G:G | Monomorphic |
| 151 | **rs72631833** | hsa-mir-183 | 100.0 | 0.0 | G:G | Monomorphic |
| 152 | **rs72646786** | hsa-mir-3972 | 99.3 | 0.114 | C:T |  |
| 153 | **rs72855836** | hsa-mir-3976 | 99.6 | 0.052 | G:A | No HWE Slovenia  No HWE total |
| 154 | **rs72996752** | hsa-mir-4999 | 95.0 | 0.249 | A:G |  |
| 155 | **rs73112689** | hsa-mir-4459 | 0.0 | 0.0 | T:T | Genotyping failure |
| 156 | **rs7311975** | hsa-mir-1178 | 99.4 | 0.041 | T:C |  |
| 157 | **rs73147065** | hsa-mir-647 | 0.0 | 0.0 | T:T | Genotyping failure |
| 158 | **rs73177830** | hsa-mir-4532 | 0.0 | 0.0 | T:T | Genotyping failure |
| 159 | **rs73235381** | hsa-mir-548h-4 | 0.0 | 0.0 | T:T | Genotyping failure |
| 160 | **rs73239138** | hsa-mir-1269a | 99.4 | 0.247 | G:A |  |
| 161 | **rs73410309** | hsa-mir-4739 | 0.0 | 0.0 | T:T | Genotyping failure |
| 162 | **rs74428911** | hsa-mir-4474 | 99.9 | 0.01 | G:T | No HWE Spain  No HWE total |
| 163 | **rs74469188** | hsa-mir-6504 | 85.7 | 0.122 | T:C |  |
| 164 | **rs745666** | hsa-mir-3615 | 99.6 | 0.364 | C:G |  |
| 165 | **rs74704964** | hsa-mir-518d | 85.8 | 0.035 | C:T |  |
| 166 | **rs74904371** | hsa-mir-2682 | 99.6 | 0.029 | C:T |  |
| 167 | **rs74949342** | hsa-mir-5702 | 100.0 | 0.006 | C:G |  |
| 168 | **rs7500280** | hsa-mir-4719 | 0.0 | 0.0 | T:T | Genotyping failure |
| 169 | **rs75019967** | hsa-mir-4477a | 99.9 | 0.0 | A:A | Monomorphic |
| 170 | **rs7522956** | hsa-mir-4742 | 99.7 | 0.233 | A:C |  |
| 171 | **rs75598818** | hsa-mir-520f | 99.7 | 0.027 | G:A |  |
| 172 | **rs75715827** | hsa-mir-944 | 99.6 | 0.071 | T:C |  |
| 173 | **rs75966923** | hsa-mir-4298 | 100.0 | 0.029 | C:A |  |
| 174 | **rs76481776** | hsa-mir-182 | 99.3 | 0.086 | C:T |  |
| 175 | **rs76800617** | hsa-mir-4521 | 100.0 | 0.023 | A:G |  |
| 176 | **rs77055126** | hsa-mir-1303 | 0.0 | 0.0 | T:T | Genotyping failure |
| 177 | **rs7709117** | hsa-mir-4634 | 98.6 | 0.446 | A:G |  |
| 178 | **rs77639117** | hsa-mir-576 | 99.4 | 0.017 | A:T |  |
| 179 | **rs78396863** | hsa-mir-4743 | 99.4 | 0.011 | G:C |  |
| 180 | **rs78541299** | hsa-mir-6075 | 100.0 | 0.003 | G:A |  |
| 181 | **rs78790512** | hsa-mir-6083 | 100.0 | 0.175 | G:A |  |
| 182 | **rs78831152** | hsa-mir-4789 | 99.7 | 0.091 | C:T |  |
| 183 | **rs78832554** | hsa-mir-4786 | 99.9 | 0.024 | G:A |  |
| 184 | **rs7896283** | hsa-mir-4481 | 67.4 | 0.404 | A:G | Genotyping failure |
| 185 | **rs7911488** | hsa-mir-1307 | 2.2 | 0.406 | A:G | Genotyping failure |
| 186 | **rs79397096** | hsa-mir-597 | 100.0 | 0.015 | G:A |  |
| 187 | **rs79512808** | hsa-mir-3976 | 100.0 | 0.013 | T:G |  |
| 188 | **rs80128580** | hsa-mir-5707 | 100.0 | 0.026 | G:A |  |
| 189 | **rs8054514** | hsa-mir-3176 | 99.9 | 0.146 | T:G |  |
| 190 | **rs8078913** | hsa-mir-4520a | 94.4 | 0.447 | C:T |  |
| 191 | **rs832733** | hsa-mir-4698 | 0.0 | 0.0 | T:T | Genotyping failure |
| 192 | **rs850108** | hsa-mir-550a-3 | 0.0 | 0.0 | T:T | Genotyping failure |
| 193 | **rs8667** | hsa-mir-4751 | 93.7 | 0.374 | G:A |  |
| 194 | **rs877722** | hsa-mir-4671 | 100.0 | 0.125 | A:T |  |
| 195 | **rs895819** | mir-27a | 2.5 | 0.167 | T:C | Genotyping failure |
| 196 | **rs897984** | hsa-mir-4519 | 0.0 | 0.0 | T:T | Genotyping failure |
| 197 | **rs9295535** | hsa-mir-5689 | 0.0 | 0.0 | T:T | Genotyping failure |
| 198 | **rs936581** | hsa-mir-3141 | 99.6 | 0.169 | G:A |  |
| 199 | **rs9842591** | hsa-mir-5186 | 85.9 | 0.457 | C:A |  |
| 200 | **rs9877402** | hsa-mir-5680 | 85.7 | 0.052 | A:G |  |
| 201 | **rs9913045** | hsa-mir-548h-3 | 0.0 | 0.0 | T:T | Genotyping failure |
| 202 | **seq_rs11048315** | MIR4302 | 99.4 | 0.127 | G:A | No HWE Spain  No HWE total |
| 203 | **seq_rs111803974** | MIR3908 | 0.0 | 0.0 | T:T | Genotyping failure |
| 204 | **seq_rs111906529** | MIR299, MIR380 | 99.9 | 0.013 | T:C |  |
| 205 | **seq_rs112328520** | MIR520G | 99.2 | 0.064 | C:T |  |
| 206 | **seq_rs11269** | mir-1282 | 100.0 | 0.0 | G:G | Monomorphic |
| 207 | **seq_rs113808830** | MIR4532 | 99.4 | 0.102 | C:T |  |
| 208 | **seq_rs116932476** | hsa-mir-4479 | 99.4 | 0.008 | G:A |  |
| 209 | **seq_rs117258475** | MIR296 | 99.9 | 0.017 | G:A |  |
| 210 | **seq_rs117650137** | hsa-mir-6717 | 100.0 | 0.033 | G:A |  |
| 211 | **seq_rs117723462** | MIR3649 | 100.0 | 0.008 | T:G |  |
| 212 | **seq_rs163642** | MIR4436B2 | 0.0 | 0.0 | T:T | Genotyping failure |
| 213 | **seq_rs62571442** | MIR3689 | 98.9 | 0.43 | A:G |  |
