# Supplementary material for: Involvement of SNPs in miR-3117 and miR-3689d2 in childhood acute lymphoblastic leukemia risk
Source: Oncotarget. 2018 May 1;9(33):22907–14. doi: 10.18632/oncotarget.25144 (PMC5955428; doi:10.18632/oncotarget.25144)
Supplement: Supplementary file 3 [file oncotarget-09-22907-s003.docx]

**Supplementary Table 3: Polymorphisms in miRNAs associated with B-ALL risk in the Spanish and Slovenian population**

|  |  |  | Spanish cohort | | | | Slovenian cohort | | | | Spanish and Slovenian cohorts | | | |
| --- | --- | --- | --- | --- | --- | --- | --- | --- | --- | --- | --- | --- | --- | --- |
| Gene  Location | **SNP**  **(Position)** | **Genotype** | **N (controls)**  **(N=330)** | **N(cases)**  **(N=217)** | **OR(95%CI)** | **P** | **N (controls)**  **(N=96)** | **N(cases)**  **(N=75)** | **OR(95%CI)** | **P** | **N (controls)**  **N=426** | **N(cases)**  **N=292** | **OR(95% CI)** | **P** |
| mir3117-3p  1p31.3 | rs12402181  (seed) | GG  AG  AA | 257 (77.9)  71 (21.5)  2 (0.6) | 155 (71.4)  57 (26.3)  5 (2.3) | Additive  1.44 (1.01-2.08) | 0.047* | 75 (78.1)  19 (19.8)  2 (2.1) | 48 (64)  25 (33.3)  2 (2.7) | Dominant  2.01 (1.02-3.95) | 0.041* | 332 (77.9)  90 (21.1)  4 (0.9) | 203 (69.5)  82 (28.1)  7 (2.4) | Additive  1.53 (1.12-2.09) | 0.006* |
|  |  | G  A | 585 (88.6)  75 (11.4) | 367 (84.6)  67 (15.4) | 1.42 (0.99-2.02) | 0.050* | 169 (88)  23 (12) | 121 (80.7)  29 (19.3) | 1.76 (0.97-3.19) | 0.06 | 754 (88.5)  98 (11.5) | 488 (83.6)  96(16.4) | 1.51 (1.11-2.05) | 0.007* |
| mir3689d2  9q34.3 | rs62571442  (PM) | TT CT CC | 117 (36.2)  151 (46.7)  55 (17.0) | 60 (27.8)  114 (52.8)  42 (19.4) | Dominant  1.48 (1.02-2.15) | 0.039* | 30 (31.2)  56 (58.3)  10 (10.4) | 22 (29.3)  31 (41.3)  22 (29.3) | Recessive  3.57 (1.57-8.12) | 0.001* | 147 (35.1)  207 (49.4)  65 (15.5) | 82 (28.2)  145 (49.8)  64 (22) | Additive  1.32 (1.06-1.64) | 0.011* |
|  |  | T  C | 385 (59.6)  261 (40.4) | 234 (54.2)  198 (45.8) | 1.24 (0.97-1.59) | 0.07 | 116 (60.4)  76 (39.6) | 75 (50)  75 (50) | 1.52 (0.99-2.34) | 0.054 | 501 (59.8)  337 (40.2) | 309 (53.1)  273 (46.9) | 1.31 (1.06-1.6) | 0.012* |

Abbreviation: PM: pre-miRNA, OR: Odd ratio, CI: Confidence interval *Significant SNPs.
