# Supplementary material for: Involvement of SNPs in miR-3117 and miR-3689d2 in childhood acute lymphoblastic leukemia risk
Source: Oncotarget. 2018 May 1;9(33):22907–14. doi: 10.18632/oncotarget.25144 (PMC5955428; doi:10.18632/oncotarget.25144)
Supplement: Supplementary file 4 [file oncotarget-09-22907-s004.docx]

**Supplementary Table 4**: Polymorphisms in miRNAs associated with B-ALL risk in the Spanish cohort

| **Gene**  **(Location)** | **SNP**  **(Position)** | **Genotype** | **N (controls)**  **(N=330)** | **N(cases)**  **(N=217)** | **OR(IC 95%)** | ***P*** | ***P*-value adjusted**  **by sex** |
| --- | --- | --- | --- | --- | --- | --- | --- |
| **Mir3166**  **11q14.2** | rs35854553  (PM) | AA AT TT | 232 (83.2)  44 (15.8)  3 (1.1) | 183 (93.4)  13 (6.6)  0 | Dominant  0.35 (0.18-0.67) | 0.0006 | 0.001 |
| **Mir3144**  **6q22.31** | rs68035463  (PM) | CC AC AA | 209 (63.7)  107 (32.6)  12 (3.7) | 115 (53.5)  82 (38.1)  18 (8.4) | Additive  1.51(1.13-2.01) | 0.004 | 0.004 |
| **Mir4745**  **19p13.3** | rs10422347  (M) | CC CT TT | 283 (87.3)  40 (12.3)  1 (0.3) | 166 (78.3)  44 (20.8)  2 (0.9) | Dominant  1.91 (1.2-3.04) | 0.005 | 0.004 |
| **Mir5196**  **19q13.12** | Rs10406069  (PM) | GG  AG  AA | 208 (63.8)  103 (31.6)  15 (4.6) | 123 (56.9)  90 (41.7)  3 (1.4) | Codominant  1.48 (1.03-2.12)  0.34 ((0.1-1.19) | 0.010 | 0.010 |
| **Mir612**  **11q13.1** | rs12803915  (PM) | GG AG AA | 213 (65.1)  104 (31.8)  10 (3.1) | 161 (74.2)  54 (24.9)  2 (0.9) | Additive  0.65(0.44-0.95) | 0.012 | 0.019 |
| **Mir300**  **14q32.31** | rs12894467  (PM) | CC CT TT | 148 (45.0)  141 (42.9)  40 (12.2) | 75 (34.7)  114 (52.8)  27 (12.5) | Dominant  1.54 (1.08-2.19) | 0.016 | 0.015 |
| **Mir595**  **7q36.3** | rs4909237  (PM) | CC CT TT | 233 (70.8)  90 (27.4)  6 (1.8) | 145 (67.1)  59 (27.3)  12 (5.6) | Recessive  3.17 (1.17-8.57) | 0.018 | 0.019 |
| **Mir4653**  **7q22.1** | rs11983381  (PM) | AA AG GG | 227 (69)  96 (29.2)  6 (1.8) | 121 (61.1)  68 (34.3)  9 (4.5) | Additive  1.43 (1.04-1.98) | 0.029 | 0.029 |
| **Mir-2278**  **9q22.32** | rs356125  (PM) | GG AG AA | 291 (88.2)  36 (10.9)  3 (0.9) | 202 (93.5)  14 (6.5)  0 | Dominant  0.52 (0.27-0.98) | 0.034 | 0.036 |
| **Mir4308**  **14q22.3** | Rs28477407  (PM) | CC  CT  TT | 281 (85.2)  46 (13.9)  3 (0.9 | 172 (79.3)  45 (20.7)  0 | Codominant  1.6(1.02-2.5)  0 | 0.037 | 0.025 |
| **Mir3689d2**  **9q34.3** | rs62571442  (PM) | AA AG GG | 117 (36.2)  151 (46.7)  55 (17.0) | 60 (27.8)  114 (52.8)  42 (19.4) | Dominant  1.48 (1.02-2.15) | 0.039 | 0.043 |
| **Mir4432** | rs243080  (PM) | CC CT TT | 98 (29.8)  175 (53.2)  56 (17) | 69 (32.9)  90 (42.9)  51 (24.3) | Recessive  1.56 (1.02-2.4) | 0.040 | 0.036 |
| **Mir3683**  **7p22.1** | rs6977967  (PM) | AA AG GG | 201 (60.9)  113 (34.2)  16 (4.8) | 150 (69.4)  57 (26.4)  9 (4.2) | Dominant  0.69(0.48-0.99) | 0.040 | 0.047 |
| **Mir4634**  **5q35.2** | rs7709117  (PM) | AA AG GG | 104 (31.7)  155 (47.3)  69 (219 | 50 (23.7)  123 (58.3)  38 (18) | Dominant  1.49 (1.01-2.22) | 0.042 | 0.040 |
| **Mir3117**  **1p31.3** | rs12402181  (seed) | GG  AG  AA | 257 (77.9)  71 (21.5)  2 (0.6) | 155 (71.4)  57 (26.3)  5 (2.3) | Additive  1.44 (1.01-2.08) | 0.047 | 0.047 |
