# Supplementary material for: Involvement of SNPs in miR-3117 and miR-3689d2 in childhood acute lymphoblastic leukemia risk
Source: Oncotarget. 2018 May 1;9(33):22907–14. doi: 10.18632/oncotarget.25144 (PMC5955428; doi:10.18632/oncotarget.25144)
Supplement: Supplementary file 5 [file oncotarget-09-22907-s005.docx]

**Supplementary Table 5**: Polymorphisms in miRNAs associated with B-ALL risk in the Slovenian cohort

| **Gene**  **(Location)** | **SNP**  **(Position)** | **Genotype** | **N (controls)**  **(N=96)** | **N(cases)**  **(N=79)** | **OR(CI 95%)** | ***P*** | ***P*-value adjusted by sex** |
| --- | --- | --- | --- | --- | --- | --- | --- |
| **Mir3972**  **1p36.13** | rs72646786  (PM) | CC CT TT | 70 (72.9)  25 (26)  1 (1) | 68 (91.9)  4 (5.4)  2 (2.7) | Dominant  0.24 (0.09-0.61) | 0.0011 | 0.0009 |
| **Mir3689d2**  **9q34.3** | rs62571442  (PM) | AA AG GG | 30 (31.2)  56 (58.3)  10 (10.4) | 22 (29.3)  31 (41.3)  22 (29.39 | Recessive  3.57 (1.57-8.12) | 0.0016 | 0.003 |
| **Mir5189**  **16q24.2** | rs56292801  (PM) | GG AG AA | 51 (53.1)  41 (42.7)  4 (4.2) | 33 (44.6)  27 (36.5)  14 (18.9) | Recessive  5.37 (1.69-17.08) | 0.0017 | 0.001 |
| **Mir4293**  **10p13** | rs12780876  (PM) | TT AT AA | 28 (29.2)  54 (56.2)  14 (14.6) | 36 (48.6)  35 (47.3)  3 (4.1) | Additive  0.45 (0.27-0.76) | 0.0018 | 0.003 |
| **Mir5189**  **16q24.2** | rs35613341  (PM) | CC CG GG | 49 (51)  41 (42.7)  6 (6.2) | 29 (39.2)  30 (40.5)  15 (20.3) | Recessive  3.81 (1.4-10.39) | 0.005 | 0.004 |
| **Mir3175**  **15q26.1** | rs1439619  (PM) | AA AC CC | 34 (35.4)  41 (42.7)  21 (21.9) | 8 (16)  23 (46)  19 (38) | Additive  1.93 (1.19-3.11) | 0.006 | 0.005 |
| **Mir5682**  **3q13.33** | rs9877402  (PM) | AA AG | 88 (93.6)  6 (6.4) | 39 (78)  11 (22) | Codominant  4.14 (1.43-11.9) | 0.007 | 0.006 |
| **Mir3615**  **17q25.1** | rs745666  (PM) | CC CG GG | 40 (41.7)  39 (40.6)  17 (17.7) | 30 (40)  41 (54.7)  4 (5.3) | Recessive  0.27 (0.08-0.81) | 0.010 | 0.010 |
| **Mir4772**  **2q12.1** | rs62154973  (M) | CC  CT  TT | 69 (71.9)  27 (28.1)  0 | 60 (83.3)  10 (13.9)  2 (2.8) | Codominant  0.43 (0.19-0.95)  0 | 0.018 | 0.025 |
| **Mir4520-1**  **17p13.1** | rs8078913  (PM) | CC CT TT | 28 (32.2)  41 (47.1)  18 (20.7) | 10 (16.7)  31 (51.7)  19 (31.7) | Additive  1.7 (1.06-2.74) | 0.026 | 0.019 |
| **Mir3166**  **11q14.2** | rs35854553  (PM) | AA  AT | 86 (89.6)  10 (10.4) | 38 (76)  12 (24) | Codominant  2.72 (1.08-6.83) | 0.033 | 0.035 |
| **Mir548AL**  **11q13.4** | rs515924  (seed) | AA AG GG | 74 (77.1)  21 (21.9)  1 (1.0) | 46 (62.2)  27 (36.5)  1 (1.4) | Dominant  2.05 (1.05-4) | 0.034 | 0.033 |
| **Mir3117**  **1p31.3** | rs12402181  (seed) | GG AG AA | 75 (78.1)  19 (19.8)  2 (2.1) | 48 (64)  25 (33.3)  2 (2.7) | Dominant  2.01 (1.02-3.95) | 0.041 | 0.039 |
